# Commercial processed soy-based food product contains glycated and glycoxidated lunasin proteoforms

**DOI:** 10.1038/srep26106

**Published:** 2016-05-18

**Authors:** Aida Serra, Xavier Gallart-Palau, Rachel Su-En See-Toh, Xinya Hemu, James P. Tam, Siu Kwan Sze

**Affiliations:** 1 School of Biological Sciences, Nanyang Technological University, 60 Nanyang Drive, 637551 Singapore

## Abstract

Nutraceuticals have been proposed to exert positive effects on human health and confer protection against many chronic diseases. A major bioactive component of soy-based foods is lunasin peptide, which has potential to exert a major impact on the health of human consumers worldwide, but the biochemical features of dietary lunasin still remain poorly characterized. In this study, lunasin was purified from a soy-based food product via strong anion exchange solid phase extraction and then subjected to top-down mass spectrometry analysis that revealed in detail the molecular diversity of lunasin in processed soybean foods. We detected multiple glycated proteoforms together with potentially toxic advanced glycation end products (AGEs) derived from lunasin. In both cases, modification sites were Lys24 and Lys29 located at the helical region that shows structural homology with a conserved region of chromatin-binding proteins. The identified post-translational modifications may have an important repercussion on lunasin epigenetic regulatory capacity. Taking together, our results demonstrate the importance of proper chemical characterization of commercial processed food products to assess their impact on consumer’s health and risk of chronic diseases.

Many epidemiological studies have demonstrated that nutritional modification can reduce the prevalence of major chronic disorders including diabetes and coronary heart diseases^1^,^2^,^3^. The health benefits of nutritional modification have been widely attributed to the influence of bioactive dietary compounds known as ‘nutraceuticals’, whose range of biological effects are only now being fully uncovered. While nutraceutical peptides are commonly found in many different foodstuffs including eggs and plants^4^,^5^,^6^, to the best of our knowledge their potential to influence human health and immunity remain poorly understood. This is in part due to the lack of biochemical characterization of dietary peptides, which limits attempts to predict their likely impact on human cells and tissues *in vivo*.

The biological activities of proteins and peptides are regulated by enzymatic and spontaneous post-translational modifications (PTMs) to amino acid side chains^7^. These PTMs change protein structure and function, resulting in the molecular diversification of individual gene products^8^. Common PTMs include the enzymatic addition of monosaccharides or extended sugar chains to the core protein (glycosylation), and the non-enzymatic addition of glycans caused by Maillard reactions between reducing sugars and amino functional groups (glycation). Covalent addition of sugar moieties to proteins has previously been documented to yield food products with antioxidant and antibiotic properties^9^,^10^. However, oxidation of Maillard reaction derivatives can also result in the formation of advanced glycation end products (AGEs), which contribute to the pathophysiology of major human disorders, including diabetes^11^ and Alzheimer’s disease^12^, by increasing oxidative stress^13^ and inflammation^14^, which both can promote cancer^15^.

In biological systems, production of AGEs occurs under hyperglycemic conditions as a response to cellular stress^16^. In particular, the AGEs *N*ε-carboxy-methyl-lysine (CML) and *N*ε-carboxy-ethyl-lysine (CEL) have been the subject of intensive study due to their ability to interact with the human cell receptor for AGEs (RAGE)^17^. However, synthesis of AGEs is not restricted to *in vivo* environments, since these compounds can also be formed during food production. The concentration and diversity of AGEs varies between different foodstuffs, being more abundant in animal-derived products and heat-processed foods^18^,^19^,^20^,^21^, while techniques such as roasting, frying and searing can also promote AGEs formation^18^,^22^. It is clear therefore that diet is a major component of human exposure to AGEs^23^ and consumption of AGEs will vary significantly between different populations around the world.

For generations, Asian populations have consumed diets that are high in soy, which is thought to contribute to the low relative risk of disorders such as osteoporosis and cardiovascular disease in this group^24^,^25^,^26^. In contrast, soybean has only recently been introduced into Western diets, where it has been consumed in a largely processed state. Despite the virtues historically attributed to soybean intake mainly credited to the presence of isoflavones and their effect on breast cancer^27^,^28^, multiple studies have reported null association of soybean intake and risk of breast cancer^29^,^30^,^31^, or even harmful effect (Hirose, *et al.*^32^ showed an increased risk of breast cancer with consumption of *atsuage*, (deep fried tofu) in postmenopausal women). Similarly, harmful associations were also observed between soybean intake and other types of cancers. A clear example is the epidemiological study performed by Sun, *et al.*^33^ with Chinese Singaporeans to evaluate the relation between the intake of soybean and the risk of bladder cancer, where 329,848 person-years of follow-up were accumulated. From that study a 2.3-fold increase in cancer risk was observed for the highest quartile of total soy intake (≥92.5 g/1000 kcal) after controlling for smoking habits and education. Based on this set of divergent studies, we could then affirm that the effect of soybean intake on health is still far to be fully elucidated. Presence and interaction of other nutraceuticals have to be considered when studying the effect of soybean intake on health^33^. Therefore thorough understanding the impact on health can only be achieved after comprehensive chemical characterization of soybean nutraceuticals^33^.

A key nutraceutical of soybean is the peptide lunasin, which is found in varying concentrations in different soy-based foods, and has been reported to exert a wide range of biological effects^34^,^35^,^36^,^37^, emphasizing its strong epigenetic regulatory capacity (as reviewed by Hernández-Ledesma and de Lumen^38^), we therefore sought to use a top-down proteomics strategy to identify and characterize novel proteoforms of lunasin from commercial soybean food products, with the ultimate aim of shedding new light on the likely biological effects of these proteoforms following human consumption. Top-down proteomics - first introduced by McLafferty and co-workers - employs state of the art mass spectrometry (MS) technology to characterize PTMs in intact proteins^39^,^40^,^41^,^42^. This approach is uniquely capable of detecting multiple proteoforms in complex samples using fragmentation by electron capture dissociation (ECD), collision activated dissociation (CAD) and electron transfer dissociation (ETD)^43^,^44^. In the current report, we used an Orbitrap Elite mass spectrometer to identify that dietary lunasin exists as a heterogeneous mixture of glycated and glycoxidated variants likely to exert distinct biological effects *in vivo*. The top-down proteomic approach used in this study also allowed us to characterize the modification sites to uncover lunasin diversity in a soy-based foodstuff. These data improve our current understanding of the nutraceutical content of soy-based foods and will assist future analyses of how lunasin ingestion impacts on human health and risk of disease.

## Results and Discussion

### Identification of lunasin in soybean products

**Figure 1:**
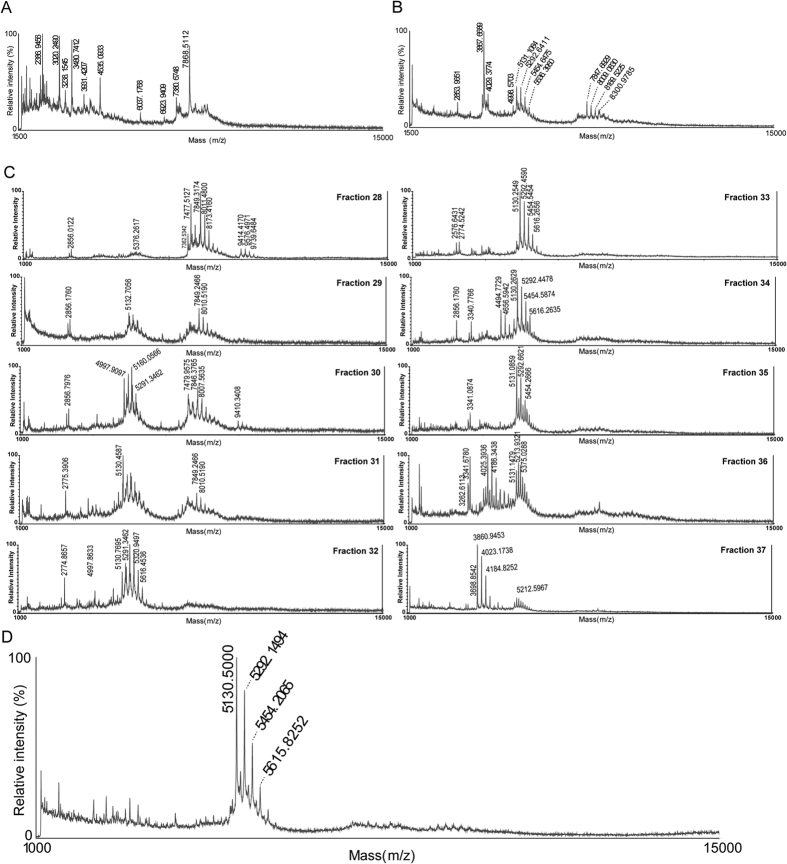
Analysis of soybean product extract by linear-mode MALDI-TOF MS. (**A**) Spectrum of raw soybean extract. No peaks were detected in the 5000–6000 m/z mass range. (**B**) Spectrum of commercial soybean beverage powder extract. Multiple peaks with laddering mass-shift of 162.05 Da were identified in 5000–6000 m/z. (**C**) Lunasin-containing fractions obtained by reversed-phase high-performance liquid chromatography (RP-HPLC). (**D**) Spectrum of purified lunasin obtained by strong anion exchange solid phase extraction (SAX-SPE).

The soybean-derived peptide lunasin is thought to exert a range of biological effects that could be modified by protein structural changes during commercial processing. We therefore sought to determine the structural diversity of lunasin peptides in soy-based foodstuffs to investigate their potential impact on human health. To do this, we used a top-down proteomics approach to study the characteristics of lunasin peptide derived from two different dietary sources – raw soybeans and commercial soybean beverage powder. Matrix-assisted laser desorption ionization-time of flight mass spectrometry (MALDI-TOF MS) analysis revealed that while the analyzed raw soybean extract contained little or no lunasin, the soybean beverage powder extract displayed a cluster of peptides in the 5000 Da mass range, suggesting the presence of multiple lunasin variants in this product (Fig. 1A,B). Moreover, the cluster of peaks detected in soybean beverage powder extract displayed a laddering mass-shift of 162 Da, indicating the presence of PTMs in these peptides.

Consistent with previous reports, our data indicated that raw soybean extract contained only trace levels of lunasin, whereas the same peptide is relatively abundant and appears structurally diverse in commercial soy-based foods^45^. This discrepancy may be due to the use of different soybean genotypes^46^, and/or the impact of environmental factors during plant development^36^. In addition, the amount of lunasin present in soybean seeds increases with maturation and decreases during sprouting^47^. While it is common for commercially processed soybean to be subjected to periods of prolonged soaking, which could perhaps promote lunasin degradation in the seeds, our analysis indicated that dried raw soybean was not enriched in lunasin compared with the soaked beans (data not shown).

### Purification of lunasin from soybean beverage powder

**Figure 2:**
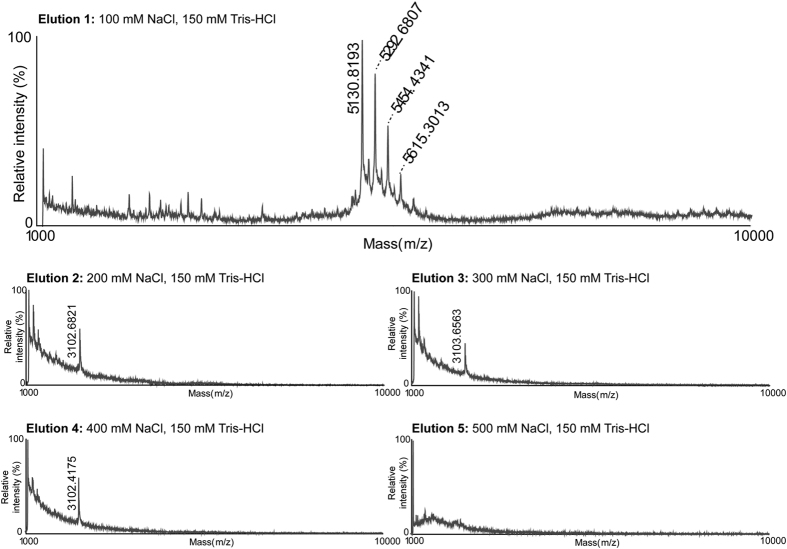
Optimization of the elution step for lunasin purification by strong anion exchange solid phase extraction (SAX-SPE). MALDI-MS spectra of samples eluted sequentially using varying concentrations of salt (100–500 mM NaCl, 150 mM Tris-HCl). Lunasin and its modified variants were completely eluted at 100 mM NaCl, 150 mM Tris-HCl.

To confirm the presence of lunasin in soybean beverage powder, we next tested two different strategies for purifying the peptide from crude extract; reverse phase high pressure liquid chromatography (RP-HPLC) and strong anion exchange solid phase extraction (SAX-SPE). For RP-HPLC purification, lunasin separation from other proteinaceous compounds was optimized by modifying the HPLC gradient. MALDI-TOF MS analysis of the fractions collected revealed that lunasin was eluted over several different fractions and partially co-eluted with other proteins. Fraction numbers 32 and 33 contained the purest lunasin (Fig. 1C). For SAX-SPE purification, we achieved efficient isolation of lunasin using salt-containing phases (Fig. 1D). Optimization of the SAX-SPE strategy was performed by testing elution efficacy using increasing concentrations of NaCl (100–500 mM), Tris-HCl (150 mM) (Fig. 2). We observed that lunasin was completely eluted when using a buffer containing 100 mM NaCl, 150 mM Tris-HCl. It has been reported that other anionic sepharose exchange stationary phases showed an optimal elution of lunasin at 200–300 mM NaCl^48^. Our use of lower salt concentrations during elution improved the purity of the recovered lunasin.

While both purification techniques were capable of isolating unmodified lunasin as well as structural variants of this peptide, isolation by SAX-SPE delivered better results than did RP-HPLC. Indeed, since SAX-SPE is also relatively simple, enables rapid sample purification, and requires only small volumes of solvents and could easily be scaled up to allow processing of large sample volumes by increasing the size of the solid phase extraction cartridge. In addition, this method yielded a total of 0.56 mg of lunasin/g of soybean beverage powder (protein quantification performed by Bradford assay) which was comparable to the scalable purification presented by Seber *et al.*^49^ where 0.44 mg of lunasin/g of soybean white flake were obtained.

### Characterization of lunasin by top-down mass spectrometry

**Figure 3:**
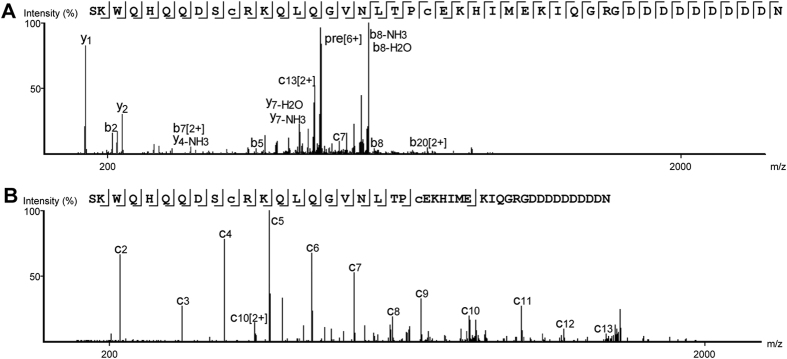
Lunasin characterization by top-down mass spectrometry. (**A**) HCD spectrum of unmodified lunasin. (**B**) ETD spectrum of unmodified lunasin (lacking *z-*type ions due to the presence of the poly-Asp-carboxyl tail).

Top-down proteomics represents a powerful tool for the characterization of proteins and peptide proteoforms by providing complete amino acid sequences and PTM profiles^39^,^40^,^41^,^42^, whereas bottom-up strategies depend on enzymatic digestion steps that disrupt the links between peptides and their parent proteoforms^41^,^50^. We therefore applied a top-down approach to characterize the lunasin proteoforms we isolated from the commercial soybean food product. Prior to MS analysis, lunasin peptide was reduced and the Cys residues converted into positively charged pseudo-Lys by alkylation in bromoethylamine (BrEA), thereby achieving a mass increase of 2 × 43.04 Da and an addition of two positive charges to improve fragmentation by ETD^43^. While we initially intended to dissolve the sample in 50% acetonitrile (ACN), 0.1% formic acid (FA) for analysis by top-down nanospray ionization tandem MS (NSI-MS/MS) (in-line with reports that acidified ~50% organic solvent is optimal for NSI top-down studies^42^), we instead observed that spectrum quality and signal strength were greater when the sample was dissolved in low organic solvent. This is likely due to the fact that lunasin contains a poly-Asp carboxyl tail at the C-terminal site of the sequence, and this region of dense negative charge may limit solubility in high organic solvent. We therefore used 3% ACN, 0.1% FA during sample preparation, which enabled us to successfully detect and fragment unmodified lunasin via high-energy collision dissociation (HCD) (Fig. 3A).

**Figure 4:**
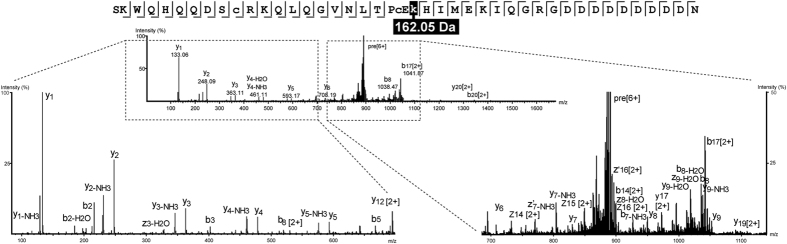
Characterization of glycated lunasin by top-down MS. HCD spectrum of single glycated lunasin with a mass addition of 162.05 Da at Lys24.

**Figure 5:**
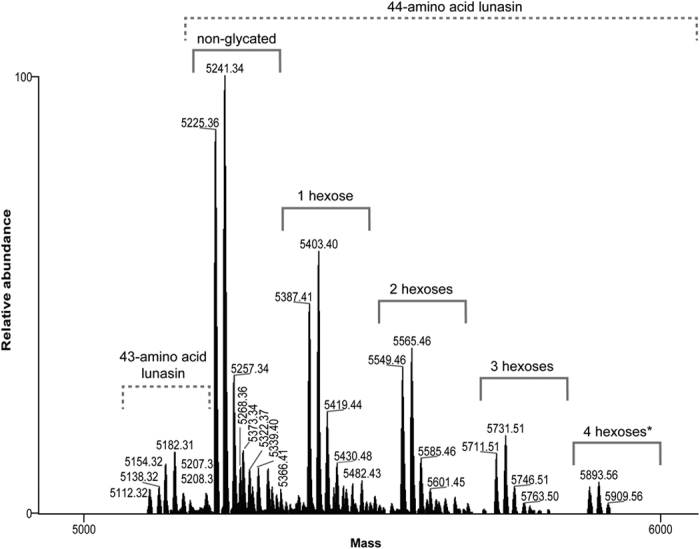
Deconvoluted full-MS spectra including all peptides detected in the mass range 5000–6000 m/z.

Lunasin is structurally defined as a 43-amino acid peptide that contains nine Asp residues at the C-terminal end^51^. However, our analysis of derivatized lunasin in soybean beverage powder revealed that the most abundant variant was a 44-amino acid peptide with a monoisotopic mass of 5225.36 Da and an extra C-terminal Asn residue (SKWQHQQDSCRKQLQGVNLTPCEKHIMEKIQGRGDDDDDDDDDN), consistent with the findings of Seber *et al.*^49^. We next proceeded to characterize lunasin structure in the soybean beverage powder extract by fragmenting the purified derivatized peptide using HCD and ETD by liquid chromatography coupled to tandem mass spectrometry (LC-MS/MS)^52^,^53^. ETD fragmentation typically generates *c-* and *z-*series ions, while collision-induced dissociation leads to the generation of *b-* and *y-*series ions^54^, hence we combined data from both of these complementary modes to robustly determine the amino acid sequence and PTM profile of lunasin peptide. Fragmentation of parent ions, which were predominantly ions at six charged state, revealed the presence of multiple lunasin proteoforms that were mainly generated by heterogeneous glycation. These post-translationally modified proteoforms were found to co-exist with the unmodified lunasin, indicating that not all the lunasin in commercial soy-based foods was altered by processing. The negatively charged poly-Asp-carboxyl tail of lunasin reduced the efficiency of ETD in the vicinity of this region of the peptide backbone (Fig. 3B). We detected distinct proteoforms of lunasin that exhibited different degrees of glycation mainly at residue Lys 24 and Lys 29. Figure 4 shows the MS spectrum of lunasin displaying a single glycation, which represented near 50% of total glycated lunasin (Fig. 5), while Supplementary information of annotated MS/MS spectra list 1 shows the spectra of lunasin proteoforms displaying either two or three sugar moieties, which represented the 30% and 14% of total glycated lunasin (Fig. 5). We were also able to detect a lunasin proteoform corresponding to the addition of four sugar moieties (4 × 162.05 Da), about 6% of the total of glycated lunasin, and we detected also multiple non-glycated proteforms derived from the 43-amino acid lunasin (Fig. 5), but it was not possible to successfully sequence these species by MS/MS due to its low abundance in the sample.

Further top-down characterization of lunasin also revealed the occurrence of alternative side chain modifications (Table 1), including oxidation (Met), dihydroxy (Lys), dehydration (Asp/Asn), deamidation (Asn/Gln), methyl esterification (Asp), carbamylation (Lys), acetylation (Lys) and pyro-glutamate conversion (N-terminal Gln) (annotated MS spectra are shown in Supplementary information of annotated MS/MS spectra list 1). In general, these PTMs were detected at lower concentrations than glycations, except for oxidation which was more frequently observed (based on the relative intensity of the parent ions shown in the full MS spectrum, Fig. 5; and the number of spectra identified, Table 1). Certain peptide/protein PTMs can occur via spontaneous chemical reaction, such as oxidation, deamidation, dehydration and pyro-glutamate conversion. Other PTMs may occur naturally in plants e.g. methyl esterification of aspartyl residues in seeds mediated by the protein-repair enzyme L-isoaspartyl methyltransferase^55^. While it remains poorly understood how peptide modification results in the addition of dihydroxylysine (+31.99 Da), previous studies have identified this PTM in the primitive vertebrate antimicrobial peptide styelin D^56^, and in an antibiotic dipeptide derived from bacteria^57^. The molecular diversities generated via enzymatic or non-enzymatic PTMs are likely to significantly modify the bioactivities of lunasin.

It is established that glycation can modulate the bioactivities of specific peptides. For example, the insect-derived antibiotic peptide drosocin enhances its bioactivity almost 100-fold when it becomes glycosylated^10^, and glycated pea albumin exhibits increased capacity to modulate the composition of gut bacteria during culture *in vitro*^58^. Our data now suggest that nutraceutical peptides in soy-based foodstuffs also exhibit multiple glycated variants with potential to exert a range of biological effects *in vivo*. Nonetheless, the complex system of secondary reactions that may follow the simple initial reaction between sugars and proteinaceous giving rise to the final production of AGEs situates glycated peptides in a controversial position.

### Advanced glycation end products derived from lunasin

While consumption of glycated bioactive peptides may confer significant health benefits, dehydration, condensation and atom rearrangement of early glycation products led to the formation of AGEs^59^. Dietary AGEs are readily absorbed via gut and can accumulate in body tissues where they contribute to the progression of several different disorders including diabetes, atherosclerosis and kidney disease^60^,^61^,^62^,^63^. We therefore probed for the presence of the well-characterized AGEs CEL and CML^17^ in the commercial soybean derivative studied here (Table 2).

AGEs from food are generally characterized by fluorimetric assays, whether by enzyme-linked immunosorbent assay based on an anti-CML monoclonal antibody^18^ or by fluorescence spectroscopy couple to a high-performance liquid chromatography^64^. The use of the top-down proteomic platform allowed us to go further in the characterization of AGEs derived from lunasin providing modification site information as well as CEL/CML identification.

We detected a total of six different lunasin-derived AGEs in the commercial soybean beverage powder, where four of them were derived from the 43-amino acid parent peptide. In contrast, we detected only two low abundant AGE-modified sequences derived from the 44-amino acid lunasin (see Supplementary information of annotated MS/MS spectra list 2 for the annotated spectra). The most abundant AGE derived from lunasin (based on spectral count) contained both CML and CEL modifications at Lys 24 and Lys 29, respectively. AGEs derived from lunasin present in the commercial soybean beverage powder were presumably generated spontaneously during food processing which involved thermal processing of the product, although water based culinary methods tend to be milder methods for the generation of AGEs in foods^65^ (technical parameters of the production process were not available).

### Longitudinal study of lunasin glycated and glycoxidated proteoforms

**Figure 6:**
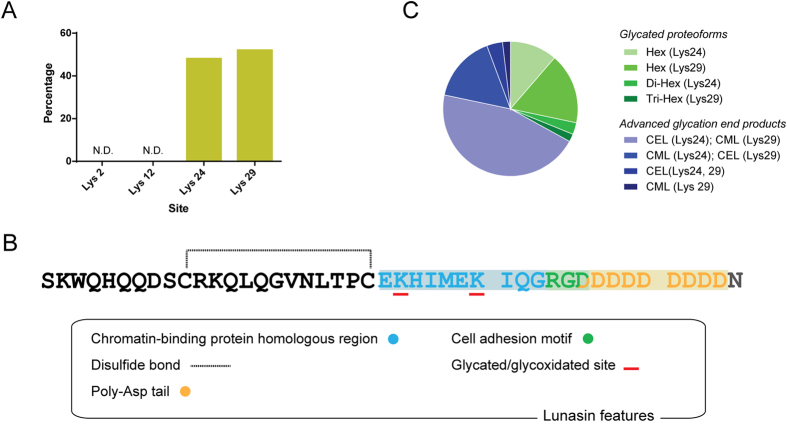
Glycated/glycoxidated modification sites in lunasin sequence. (**A**) Occurrence of glycation and glycoxidation at the four Lys present in lunasin sequence. N.D. means not detected. (**B**) Description of distinct features present in lunasin sequence and localization of glycated/glycoxidated sites. Modified Lys residues (underlined in red) are both located at the chromatin-binding protein homologous region of lunasin (shaded in blue). (**C**) Analysis of the diversity of glycation and glycoxidation species detected at the residues Lys 24 and Lys 29. Distribution was calculated considering both analyzed batch and based on total spectral counts.

We evaluated the occurrence of glycation/glycoxidation in lunasin peptide from commercial soybean beverage powder by performing a longitudinal study. To achieve this aim we profiled lunasin isolated from two independent batches of product manufactured in a three months interval. We observed that glycation and glycoxidation sites remained constant at residues Lys 24 and Lys 29 of lunasin whereas no glycation/glycoxidation was observed at residues Lys 2 and Lys 12 (Table 2 and Fig. 6A). Intriguingly, Lys 24 and Lys 29 are located at the helical region (EKHIMEKIQG) that shows structural homology with a conserved region of chromatin-binding proteins^38^ (Fig. 6B). Because this region is involved in targeting of H3-H4 histones^66^, it might be a highly exposed region of lunasin sequence. We hence explain the fixed localization of glycation/glycoxidation at Lys 24 and Lys 29 as dependent of the peptide conformation while the direct effect of production process is evidenced on the highly diverse glycated (mono-, di- and tri-hexose) and glycoxidated (CML/CEL) products detected (Fig. 6C). Presence of glycation/glycoxidation in that key region of the sequence may then have an important repercussion on lunasin epigenetic regulatory capacity, thus further studies have to be carried out to elucidate the impact of PTMs.

We further compared the presence of glycated and glycoxidated lunasin from both batches of product. This analysis revealed that the amount of AGEs in commercial soybean beverage powder ranged between 68.9 and 183.7 μg/g. Content of AGEs in foods depends mainly on food matrix and food processing^18^,^64^. Nonetheless, hazardousness of AGEs not only depends on the amount of AGEs consumed, but it also depends on which molecules and which sites are modified. Besides, non-modified lunasin ranged between 0.22 and 0.23 mg/g and glycated lunasin ranged between 27.1 and 38.8 μg/g. (Table 3). We hypothesize that these variations could be consequence of the complexity of the chemical reactions that took place during food processing.

## Materials and Methods

### Soybean products

Raw soybeans and commercial soybean beverage powder produced from non-genetically modified soybeans were obtained from a local supermarket in Singapore. The soybean beverage powder comprised listed ingredients of soybeans with bean coats removed and calcium carbonate added. The packaging indicated that no sugar was added to the product. Soybean beverage powder did not include other proteins from different sources. Production process described by the manufacturer combined steaming, coat removal, freeze-drying and grinding steps.

### Preparation of soybean extract

#### Extraction

Soybean extract was prepared according to the protocol described by Seber *et al.*^49^, except for minor modifications. In brief, a total of 10 g raw soybeans were soaked overnight in distilled water and then blended for 5 min together with 100 mL extraction buffer (75.5 mM sodium phosphate, 68.4 mM sodium chloride, 10 mM sodium metabisulfite, 20 mM ascorbic acid, pH 7.4). The mixture was then centrifuged at 4000 × *g* for 30 min at 4 °C. In parallel, a total of 10 g commercial soybean beverage powder was combined with 100 mL extraction buffer, shaken for 30 min, and then centrifuged at 4000 × *g* for 30 min at 4 °C. The supernatants were collected and filtered through Whatman^™^ grade 54 and grade 42 paper filters to remove any suspended material. All the extraction process was maintained at 4 °C.

#### Defatting

To remove lipids, the soybean extracts were combined with 100 mL hexane and shaken for 2 h, after which the hexane was removed by decanting and discarding the lower layer. The upper layer was retained and subjected to a second round of hexane-based defatting as described above, before being recovered and centrifuged at 5000 × *g* for 10 minutes at 4 °C. The resultant liquid was then filtered through 0.45 and 0.22 μm filters and stored at −20 °C.

### Purification of lunasin from soybean beverage powder by strong anion exchange solid phase extraction (SAX-SPE)

Lunasin was isolated from other positively charged species by SAX-SPE (SAX Hypersep, 50 mg, Thermo Scientific, Bremen, Germany). The phases used in the extraction were 150 mM Tris-HCl buffer at pH 9 (phase A) and phase A with 0.1 M NaCl (phase B). The sample was loaded into the cartridge at pH 8.5 (for better retention of analytes in the stationary phase) and elution was performed using 1 mL phase B.

### Chromatographic purification of lunasin from soybean beverage powder

Large proteins were removed from the soybean beverage extract by passing the samples though an Amicon 30 kDa-molecular weight cut off filter (Merck Millipore, MA, USA). Lunasin peptide was then isolated from the filtrate by RP-HPLC using a Luna column (3.6 μm, 100 mm × 4.6 mm, Phenomenex Inc, Torrance, CA, USA). Water and ACN were used as mobile phases A and B, respectively. Separation was performed using a 90-min gradient as follows: 5% B for 5 min, 5–45% B for 60 min, 45–100% B for 3 min, 100% B for 7 min, and then returned to initial conditions over 0.5 min and kept isocratic for 14.5 min thereafter. A total of 400 μL soybean beverage extract was injected and fractions were collected every minute.

### Cys-to-pseudoLys derivatization

SAX-purified peptides were reduced and alkylated as previously described^67^. Briefly, purified peptides were incubated with 30 mM dithiotreitol and 60 mM BrEA in 200 mM Tris-HCl buffer (pH 8.6) for 1 h at 55 °C. Reduction and BrEA alkylation of disulfide bonds converted Cys into pseudoLys, thereby increasing the mass of each Cys by 43.04 Da with the addition of a positive charge. Desalting was carried out using a C18 Sep-Pak column (Waters, Sep-Pak C18, 100 mg sorbent, Milford, MA., USA). Elution was performed using 1 mL 75% ACN, 0.1% FA. The eluted peptide solution was then dried overnight at room temperature in a vacuum concentrator (Eppendorf, Hamburg, Germany).

### MALDI-TOF MS

Preparation of soybean extract, lunasin purification, and Cys-to-pseudoLys derivatization were monitored using an Applied Biosystems 4800 MALDI-MS analyzer. The linear acquisition mode was used in the 1000 to 15,000 Da range with a focusing mass of 7000 Da. The matrix used was saturated α-cyano-4-hydroxycinnamic acid in 75% ACN with 0.1% trifluoroacetic acid. Desalting of samples was performed with C18 zip-tips (Millipore Corp., Billerica, MA, U.S.A.) when required. Each MALDI spot comprised 0.5 μL desalted sample and 0.5 μL matrix solution.

### Top-down analysis by NSI-MS/MS

Purified peptide was dissolved in 3% ACN, 0.1% FA and then sprayed onto the detector using a Thermo Finnigan Dynamic NSI source (Thermo Scientific Inc., Bremen, Germany) with Proxeon NanoES spray capillaries (Thermo Scientific Inc). The Top-Down NSI-MS/MS analysis of purified lunasin was performed using an Orbitrap Elite mass spectrometer (Thermo Scientific Inc.) manually tuned using the isolated peptide. The capillary temperature was set to 200 °C and the spray voltage was set to 1.50 kV. Spectra were acquired in positive mode using a mass range from 200–2000 m/z and a resolving power of 120.000 (at 400 m/z). Precursor ion target was set to 1 × 10^6^ charges for full MS and MS/MS experiments. Maximum injection time for full MS and MS/MS was set to 200 ms. Between 20–50 μscans were averaged for each spectrum. The most abundant lunasin ions at six charged state (874 m/z) were isolated with 1.5 m/z isolation window, and then fragmented by HCD using 27% normalized collision energy. Data were collected manually in positive mode using LTQ Tune Plus software (Thermo Scientific Inc.) The automatic gain control (AGC) for full MS and MS/MS was set to 1 × 10^6^.

### Top-down analysis by LC-MS/MS

Lunasin characterization by LC-MS/MS was performed using an Orbitrap Elite mass spectrometer coupled to a Dionex UltiMate 3000 UHPLC system (Thermo Scientific Inc., Bremen, Germany). The sample was sprayed using a Michrom Thermo CaptiveSpray nanoelectrospray ion source (Bruker-Michrom Inc., Auburn, USA) and the separation was performed using a reversed phase Acclaim PepMap RSL column (75 μm ID × 15 cm, 2 μm particles, Thermo Scientific). Mobile phase A was 0.1% FA in water and mobile phase B was 90% ACN, 0.1% FA. Separation of peptides was performed in a 60-min gradient of 3% B for 1 min, 3–30% B for 31 min, 30–40% B for 10 min, 40–98% B for 5 min, 98% B for 5 min, and then reverted to initial conditions over 30 s and kept isocratic for 7.5 min thereafter. Data acquisition was performed in positive mode using LTQ Tune Plus software alternating between full MS and MS/MS. Preliminary data acquisition was performed using 150–2000 m/z, 60.000 resolution (at 400 m/z) with 3 μscan averaged per spectrum. For subsequent injections, we used 120.000 resolution (at 400 m/z) and 10 μscan in order to isolate the cluster of peptides at six charged state. The AGC for full MS and MS/MS was set to 1 × 10^6^ and the reagent AGC was 1 × 10^5^. The 5 most intense ions were isolated with a 1.5 Da mass isolation window and then fragmented by HCD using 27% normalized collision energy or by ETD using reaction times over 80 ms.

### Longitudinal study of lunasin proteoforms

Two batches of commercial soybean beverage powder purchased from local supermarket and manufactured in three months interval were processed as described above. From both batches, lunasin was purified and glycations and glycoxidations were profiled.

### Data Analysis

Data from top-down NSI-MS/MS was analyzed using MASH Suite (version 1.0.0.23928, UW-Madison, U.S.A.)^68^ and further validated by manual inspection. Peak deconvolution for manual data analysis was performed using the Xtract algorithm (Thermo Scientific Inc.). Data from top-down LC-MS/MS was analyzed by ProSightPTM^69^ and PEAKS studio (version 7.0, Bioinformatics Solutions, Waterloo, Canada)^70^ against Glycine max 2S albumin pre-protein sequence. In all cases, 10 ppm MS and 0.05 Da MS/MS tolerances were used for data analysis. EA (Cys) was included as a fixed modification and additional EA was set as a variable modification to account for possible polymerization of the alkylating agent. For AGE product analysis, CML (Lys) and CEL (Lys) were added as variable modifications. A stringent false discovery rate of 0.1% was set for all searches. All sequences identified were further validated by manual inspection.

### Data Deposition

The mass spectrometry proteomics data have been deposited to the ProteomeXchange Consortium^71^ via the PRIDE partner repository with the dataset identifier PXD003064.

## Conclusions

Here in this study, the molecular diversity of lunasin from a commercial soybean derived foodstuff has been successfully depicted by applying a top-down proteomics strategy. The use of this approach allowed us to investigate the presence of PTMs and to identify for the first time AGEs derived from lunasin together with glycated proteoforms. Existence of PTMs in the helical region that shows structural homology with a conserved region of chromatin-binding proteins is likely to critically impact the epigenetic regulatory capacity of lunasin. Our results thus provide novel and valuable molecular details of lunasin that have to be considered when studying its mechanisms of action and health effects. The molecular characterization of lunasin presented in this study also demonstrated the need to conduct thorough chemical characterization of putative nutraceuticals in order to assess how best to exploit these compounds for the improvement of human health via dietary modifications.

## Additional Information

**How to cite this article**: Serra, A. *et al.* Commercial processed soy-based food product contains glycated and glycoxidated lunasin proteoforms. *Sci. Rep.* **6**, 26106; doi: 10.1038/srep26106 (2016).

## Supplementary Material

Supplementary Information

## Figures and Tables

**Table 1:** Proteoforms of lunasin identified in the commercial soybean beverage powder.

Peptide	−10logP^a^	Mass	ppm	m/z	z	#Spec^b^	Side chain modifications
SKWQHQQDS**C**RKQLQGVNLTP**C**EKHIMEKIQGRGDDDDDDDDDN	200.00	5225.36	−2.3	747.4854	7	220	EA^c^
SKWQHQQDS**C**RKQLQGV	166.07	2098.05	0.1	525.5196	4	2	EA
KWQHQQDS**C**RKQLQGVNLTP**C**EKHIMEKIQGRGDDDDDDDDDN	124.41	5138.33	−0.7	857.3945	6	10	EA
GVNLTP**C**EKHIMEKIQGRGDDDDDDDDDN	112.91	3301.41	−2.7	826.3575	4	56	EA
LQGVNLTP**C**EKHIMEKIQGRGDDDDDDDDDN	112.08	3542.55	−1.2	886.6443	4	11	EA
DS**C**RKQLQGVNLTP**C**EKHIMEKIQGRGDDDDDDDDDN	100.86	4302.92	0.8	861.5914	5	12	EA
SKWQHQQDS**C**RKQLQGV	92.20	2098.05	0.1	525.5196	4	2	EA
**Q**HQQDS**C**RKQLQGVNLTP**C**EKHIMEKIQGRGDDDDDDDDDN	76.18	4807.13	−5.1	962.4275	5	4	Pyro-Glutamate conversion (N-term Gln); EA
SKWQHQQDS**C**RKQLQGVNLTP**C**EKHI**M**EKIQGRGDDDDDDDDDN	75.56	5241.35	−2	874.5645	6	38	EA; Oxidation (Met)
SKWQHQQDS**C**RKQLQGVNLTP**C**E**K**HIMEKIQGRGDDDDDDDDDN	59.40	5387.41	−2.4	898.907	6	5	EA; Hex (Lys)
SKWQHQQDS**C**RKQLQGVNLTP**C**EKHIME**K**IQGRGDDDDDDDDDN	58.47	5267.37	3	878.9047	6	5	EA; Acetylation (Lys)
WQHQQDS**C**RKQLQGVNLTP**C**EKHIMEKIQGRGDDDDDDDDDN	55.05	5010.23	−0.2	1003.053	5	1	EA
SKWQHQQDS**C**RKQLQGVNLTP**C**EKHIME**K**IQGRGDDDDDDDDDN	54.48	5257.35	−1.3	877.2308	6	14	EA; Dihydroxy (Lys)
SKWQHQQDS**C**RKQLQGVNLTP**C**EKHIMEKIQGRGDDDDDDDD**DN**	53.17	5208.33	5.7	869.0676	6	2	EA; Dehydration (Asp); Deamidation (Asn)
SKWQHQQDSCRKQLQGVNLTP**C**E**K**HIMEKIQGRGDDDDDDDDDN	52.19	5549.46	−2.3	925.9158	6	2	EA; Di-Hex (Lys)
SKWQHQQDS**C**RKQLQGVNLTP**C**EKHIMEKI**Q**GRGD**D**DDDDDDDN	52.01	5240.36	−1.9	874.3986	6	4	EA; Deamidation (Gln); Methyl ester (Asp)
SKWQHQQDS**C**RKQLQGVNLTP**C**EKHIME**K**IQGRGDDDDDDDDDN	49.76	5387.41	−1.6	898.9077	6	25	EA; Hex (Lys)
GVNLTP**C**EKHIME**K**IQGRGDDDDDDDDDN	48.69	3344.42	5.9	837.1161	4	1	EA; Carbamylation (Lys)
GVNLTP**C**E**K**HIMEKIQGRGDDDDDDDDDN	48.53	3463.46	−0.9	866.8721	4	2	EA; Hex (Lys)
SKWQHQQDS**C**RKQLQGVNLTP**C**EKHIMEKIQGRGDDDDDDD**D**DN	46.34	5207.35	−0.1	868.8985	6	2	EA; Dehydration (Asn)
GVNLTP**C**EKHIME**K**IQGRGDDDDDDDDDN	45.05	3463.46	−0.4	866.8726	4	2	EA; Hex (Lys)
SKWQHQQDS**C**RKQLQGVNLTP**C**EKHIME**K**IQGRGDDDDDDDDDN	44.53	5268.36	5.2	879.0725	6	1	EA; Carbamylation (Lys)
SKWQHQQDS**C**RKQLQGVNLTP**C**EKHIMEKI**Q**GRGDDDDDDDDDN	43.02	5226.34	1.1	872.0652	6	3	EA; Deamidation (Gln)
SKWQHQQDS**C**RKQLQGVNLTP**C**EKHIME**K**IQGRGDDDDDDDDDN	41.85	5403.41	−0.2	901.5748	6	2	EA; Galactosyl hydroxylysine (Lys)
SKWQHQQDS**C**RKQLQGVNLTP**C**EKHIMEKIQGRGDDDDD**D**DDDN	40.76	5207.35	−4.1	868.895	6	3	EA; Dehydration (Asp)
SKWQHQQDS**C**RKQLQGVNLTP**C**EKHI**M**EKI**Q**GRGDDD**D**DDDDDN	39.34	5256.35	−0.2	877.0659	6	1	EA; Oxidation (Met); Deamidation (Gln); Methyl ester (Asp)
SKWQHQQDS**C**RKQLQGVNLTP**C**EKHIME**K**IQGRGDDDDDDDDDN	38.98	5711.52	−3.6	952.9233	6	1	EA; Tri-Hex (Lys)
SKWQHQQDS**C**RKQLQGVNLTP**C**EKHIME**K**IQGRGDDDDDDDDDN	37.42	5565.46	−2.2	928.5817	6	2	EA; Glucosylgalactosyl hydroxylysine (Lys)
SKWQHQQDS**C**RKQLQGVNLTP**C**EKHIMEKIQG**R**GDDDDDDDDDN	36.08	5257.35	−4.4	877.2281	6	1	EA; Dihydroxy (Arg)
KWQHQQDS**C**RKQLQGVNLTP**C**EKHI**M**EKIQGRGDDDDDDDDDN	30.45	5154.32	−5.4	860.0562	6	1	EA; Oxidation (Met)
GVNLTP**C**EKHIMEKIQGRGDDDDDDDDD**N**	29.98	3302.39	4.2	826.6092	4	2	EA; Deamidation (Asn)

Sequences have been identified using 2S albumin pre-protein sequence as database. PTM has been further validated manually.

^a^−10logP, p-value was converted from the linear discriminative function score by the PEAKS PTM software. A higher −10logP value indicated a more confident identification.

^b^#Spec, number of spectrums for each peptide is the result of the sum of all the spectrums identified considering all replicates.

^c^EA, means Cys-to-pseudoLys derivatized Cys.

**Table 2:** Advanced glycation end products (AGEs) and glycated proteoforms derived from lunasin identified in the two different batch of commercial soybean beverage powder.

Batch		Peptide	− 10logP^a^	Mass	ppm	m/z	z	#Spec^b^	Side chain modifications
1	Glycation								
		SKWQHQQDS**C**RKQLQGVNLTP**C**E**K**HIMEKIQGRGDDDDDDDDDN	59.40	5387.41	−2.4	898.9070	6	5	EA^c^; Hex (Lys24)
		SKWQHQQDSCRKQLQGVNLTP**C**E**K**HIMEKIQGRGDDDDDDDDDN	28.05	5549.46	−1.9	925.9161	6	1	EA; Di-Hex (Lys24)
		SKWQHQQDS**C**RKQLQGVNLTP**C**EKHIME**K**IQGRGDDDDDDDDDN	49.76	5387.41	−1.6	898.9077	6	16	EA; Hex (Lys29)
		SKWQHQQDS**C**RKQLQGVNLTP**C**EKHIME**K**IQGRGDDDDDDDDDN	38.98	5711.52	−3.6	952.9233	6	1	EA; Tri-Hex (Lys29)
	AGEs								
		SKWQHQQDS**C**RKQLQGVNLTP**C**E**K**HIME**K**IQGRGDDDDDDDDD	200.00	5241.34	0.1	874.5645	6	37	EA; CEL (Lys24); CML (Lys29)
		SKWQHQQDS**C**RKQLQGVNLTP**C**E**K**HIME**K**IQGRGDDDDDDDDD	90.21	5241.34	0.1	874.5645	6	16	EA; CML (Lys24); CEL (Lys29)
		KWQHQQDS**C**RKQLQGVNLTP**C**E**K**HIME**K**IQGRGDDDDDDDDD	81.25	5154.31	−3.3	860.0562	6	2	EA; CEL (Lys24); CML (Lys29)
		SKWQHQQDSCRKQLQGVNLTPCE**K**HIME**K**IQGRGDDDDDDDDD	45.57	5255.36	−3.1	876.8976	6	3	EA; CEL(Lys24, 29)
		SKWQHQQDS**C**RKQLQGVNLTP**C**EKHIME**K**IQGRGDDDDDDDDDN	39.28	5283.36	3.3	881.5708	6	2	EA; CML (Lys 29)
		SKWQHQQDS**C**RKQLQGVNLTP**C**E**K**HIME**K**IQGRGDDDDDDDDDN	43.46	5369.40	0.1	895.9075	6	1	EA; CEL(Lys24, 29)
2	Glycation								
		SKWQHQQDS**C**RKQLQGVNLTP**C**E**K**HIMEKIQGRGDDDDDDDDDN	63.26	5387.41	−2.4	898.9070	6	5	EA; Hex (Lys24)
		SKWQHQQDSCRKQLQGVNLTP**C**E**K**HIMEKIQGRGDDDDDDDDDN	52.19	5549.46	−2.3	925.9158	6	2	EA; Di-Hex (Lys24)
		GVNLTP**C**E**K**HIMEKIQGRGDDDDDDDDDN	49.05	3463.46	−0.9	866.8721	4	2	EA; Hex (Lys24)
		GVNLTP**C**EKHIME**K**IQGRGDDDDDDDDDN	48.58	3463.46	−0.4	866.8726	4	2	EA; Hex (Lys29)
		SKWQHQQDS**C**RKQLQGVNLTP**C**EKHIME**K**IQGRGDDDDDDDDDN	38.74	5711.52	−3.6	952.9233	6	1	EA; Tri-Hex (Lys29)
	AGEs								
		SKWQHQQDS**C**RKQLQGVNLTP**C**E**K**HIME**K**IQGRGDDDDDDDDD	147.04	5241.34	1.5	874.5645	6	9	EA; CEL (Lys24); CML (Lys29)
		SKWQHQQDS**C**RKQLQGVNLTP**C**E**K**HIME**K**IQGRGDDDDDDDDD	133.77	5241.34	3.3	874.5645	6	1	EA; CML (Lys24); CEL (Lys29)

Two different units of product bought in a three month interval were processed per separate (refered as batch 1 and 2). PTM has been further validated manually. PTM modification site for non-full sequence peptides is considered based on the position of the amino acid in the full sequence of the peptide.

^a^−10logP, p-value was converted from the linear discriminative function score by the PEAKS PTM software. A higher −10logP value indicated a more confident identification.

^b^#Spec, number of spectrums for each peptide is the result of the sum of all the spectrums identified considering all replicates.

^c^EA, means Cys-to-pseudoLys derivatized Cys.

**Table 3:** Quantitative study of glycated and glycoxidated lunasin present in commercial soybean beverage powder.

	Lunasin proteoforms		
	Non-modified (mg)	Glycated (μg)	AGEs (μg)
Batch 1	0.23	27.1	183.7
Batch 2	0.22	38.8	68.9

Quantification was performed in two different batch of commercial soybean beverage powder based on spectral counts.
