# Supplementary material for: Commercial processed soy-based food product contains glycated and glycoxidated lunasin proteoforms
Source: Sci Rep. 2016 May 18;6:26106. doi: 10.1038/srep26106 (PMC4870627; doi:10.1038/srep26106)
Supplement: Supplementary Information [file srep26106-s1.pdf]

Supplementary information

Commercial processed soy-based food product contains glycated and glycoxidated lunasin proteoforms

Aida Serra[1], Xavier Gallart-Palau[1], Rachel See-Toh Su-En[1], Xinya Hemu[1], James P. Tam[1], Siu Kwan Sze[1,*]

[1]School of Biological Sciences, Nanyang Technological University, 60 Nanyang Drive, Singapore 637551.

*** Corresponding Author:**

Siu Kwan SZE, PhD

School of Biological Sciences

Nanyang Technological University,

60 Nanyang Drive, Singapore 637551

Tel: (+65) 6514-1006

Fax: (+65) 6791-3856

Email: sksze@ntu.edu.sg

Supplemental information of annotated MS/MS spectra list 1: Annotated MS/MS spectra for all the identified peptides listed in Table 1.

The annotated MS/MS spectra of the identified peptides in the following table are showed in subsequent pages. They are arranged by the ID# in the first column of the table. Modified residues are highlighted in the sequence shown in each annotated spectrum by lowercased letters. Y-axis zoomed spectra were provided when necessary in order to provide more details about the lower intensity ions.

Numbered list of detected peptide	-10lgP[a]	Mass	ppm	m/z	z	#Spec[b]	Side chain modifications
1. SKWQHQQDS**C**RKQLQGVNLTP**C**EKHIMEKIQGRGDDDDDDDDDDN	200.00	5225.36	-2.3	747.4854	7	220	EA[c]
2. SKWQHQQDS**C**RKQLQGV	166.07	2098.05	0.1	525.5196	4	2	EA
3. KWQHQQDS**C**RKQLQGVNLTP**C**EKHIMEKIQGRGDDDDDDDDDDN	124.41	5138.33	-0.7	857.3945	6	10	EA
4. GVNLTP**C**EKHIMEKIQGRGDDDDDDDDDDN	112.91	3301.41	-2.7	826.3575	4	56	EA
5. LQGVNLTP**C**EKHIMEKIQGRGDDDDDDDDDDN	112.08	3542.55	-1.2	886.6443	4	11	EA
6. DS**C**RKQLQGVNLTP**C**EKHIMEKIQGRGDDDDDDDDDDN	100.86	4302.92	0.8	861.5914	5	12	EA
7. SKWQHQQDS**C**RKQLQGV	92.20	2098.05	0.1	525.5196	4	2	EA
**8.** **Q**HQQDS**C**RKQLQGVNLTP**C**EKHIMEKIQGRGDDDDDDDDDDN	76.18	4807.13	-5.1	962.4275	5	4	EA; Pyro-Glutamate conversion (N-term Gln)
9. SKWQHQQDS**C**RKQLQGVNLTP**C**EKHI**M**EKIQGRGDDDDDDDDDDN	75.56	5241.35	-2	874.5645	6	38	EA; Oxidation (Met)
10. SKWQHQQDS**C**RKQLQGVNLTP**C**E**K**HIMEKIQGRGDDDDDDDDDDN	59.40	5387.41	-2.4	898.907	6	5	EA; Hex (Lys)
11. SKWQHQQDS**C**RKQLQGVNLTP**C**EKHIME**K**IQGRGDDDDDDDDDDN	58.47	5267.37	3	878.9047	6	5	EA; Acetylation (Lys)
12. WQHQQDS**C**RKQLQGVNLTP**C**EKHIMEKIQGRGDDDDDDDDDDN	55.05	5010.23	-0.2	1003.053	5	1	EA
13. SKWQHQQDS**C**RKQLQGVNLTP**C**EKHIME**K**IQGRGDDDDDDDDDDN	54.48	5257.35	-1.3	877.2308	6	14	EA; Dihydroxy (Lys)
14. SKWQHQQDS**C**RKQLQGVNLTP**C**EKHIMEKIQGRGDDDDDDDDD**DN**	53.17	5208.33	5.7	869.0676	6	2	EA; Dehydration (Asp); Deamidation (Asn)
15. SKWQHQQDSCRKQLQGVNLTP**C**E**K**HIMEKIQGRGDDDDDDDDDDN	52.19	5549.46	-2.3	925.9158	6	2	EA; Di-Hex (Lys)
16. SKWQHQQDS**C**RKQLQGVNLTP**C**EKHIMEKI**Q**GRGD**D**DDDDDDDDN	52.01	5240.36	-1.9	874.3986	6	4	EA; Deamidation (Gln); Methyl ester (Asp)
17. SKWQHQQDS**C**RKQLQGVNLTP**C**EKHIME**K**IQGRGDDDDDDDDDDN	49.76	5387.41	-1.6	898.9077	6	25	EA; Hex (Lys)
18. GVNLTP**C**E**K**HIMEKIQGRGDDDDDDDDDDN	48.53	3463.46	-0.9	866.8721	4	2	EA; Hex (Lys)
19. SKWQHQQDS**C**RKQLQGVNLTP**C**EKHIMEKIQGRGDDDDDDDD**D**DN	46.34	5207.35	-0.1	868.8985	6	2	EA; Dehydration (Asn)
20. SKWQHQQDS**C**RKQLQGVNLTP**C**EKHIME**K**IQGRGDDDDDDDDDDN	44.53	5268.36	5.2	879.0725	6	1	EA; Carbamylation (Lys)
21. SKWQHQQDS**C**RKQLQGVNLTP**C**EKHIMEKIQGRGDDDDDD**D**DDDN	40.76	5207.35	-4.1	868.895	6	3	EA; Dehydration (Asp)
22. SKWQHQQDS**C**RKQLQGVNLTP**C**EKHI**M**EKI**Q**GRGDDD**D**DDDDDN	39.34	5256.35	-0.2	877.0659	6	1	EA; Oxidation (Met); Deamidation (Gln); Methyl ester (Asp)
23. SKWQHQQDS**C**RKQLQGVNLTP**C**EKHIME**K**IQGRGDDDDDDDDDDN	38.98	5711.52	-3.6	952.9233	6	1	EA; Tri-Hex (Lys)
24. KWQHQQDS**C**RKQLQGVNLTP**C**EKHI**M**EKIQGRGDDDDDDDDDDN	30.45	5154.32	-5.4	860.0562	6	1	EA; Oxidation (Met)
25. GVNLTP**C**EKHIMEKIQGRGDDDDDDDDDD**N**	29.98	3302.39	4.2	826.6092	4	2	EA; Deamidation (Asn)

1.

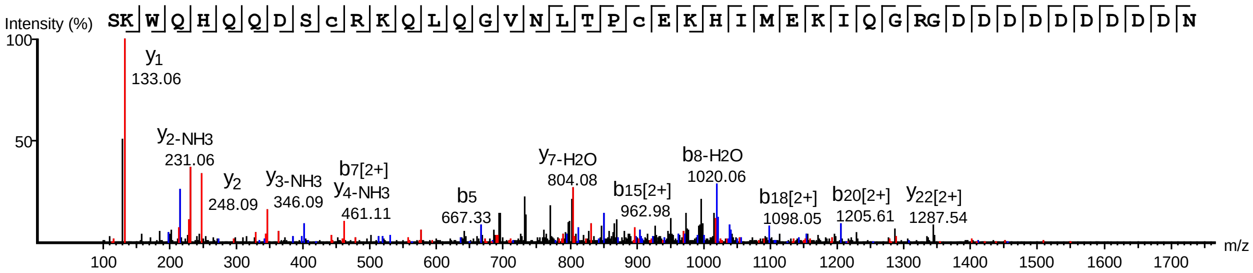
Intensity (%)
SKWQHQQDScRKQLQGVNLTPcEKHIMEKIQGRGDDDDDDDDN
100
50
y1 133.06
y2-NH3 231.06
y2 248.09
y3-NH3 346.09
b7[2+]
y4-NH3 461.11
b5 667.33
y7-H2O 804.08
b15[2+] 962.98
b8-H2O 1020.06
b18[2+] 1098.05
b20[2+] 1205.61
y22[2+] 1287.54
m/z
100 200 300 400 500 600 700 800 900 1000 1100 1200 1300 1400 1500 1600 1700

2.

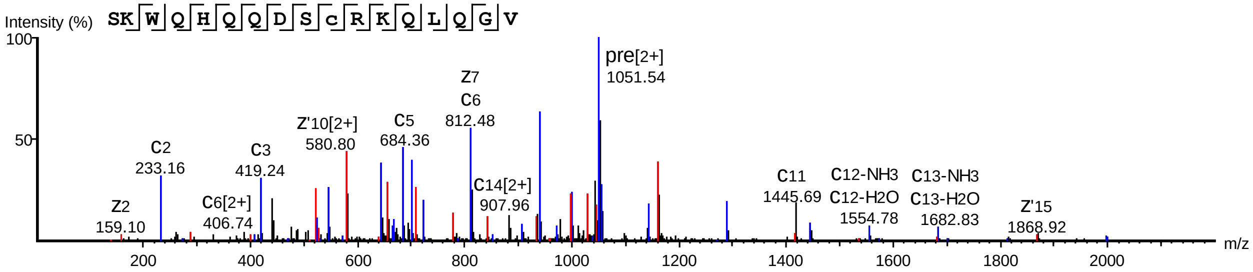
Intensity (%)
SKWQHQQDScRKQLQGV
100
50
pre[2+] 1051.54
z7
c6 812.48
z'10[2+] 580.80
c5 684.36
c2 233.16
c3 419.24
c14[2+] 907.96
c6[2+] 406.74
z2 159.10
c11 1445.69
c12-NH3
c12-H2O 1554.78
c13-NH3
c13-H2O 1682.83
z'15 1868.92
m/z
200 400 600 800 1000 1200 1400 1600 1800 2000

3.

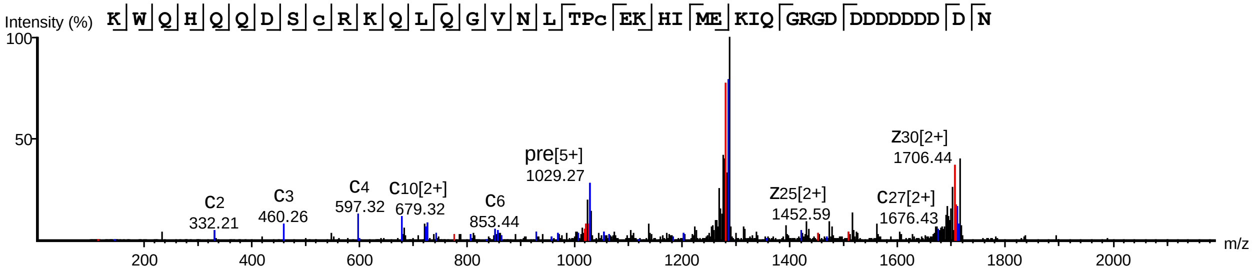
Intensity (%)
KWQHQQDScRKQLQGVNLTPcEKHIMEKIQGRGDDDDDDDDN
100
50
z30[2+] 1706.44
pre[5+] 1029.27
c4 597.32
c10[2+] 679.32
c2 332.21
c3 460.26
c6 853.44
z25[2+] 1452.59
c27[2+] 1676.43
m/z
200 400 600 800 1000 1200 1400 1600 1800 2000

4.

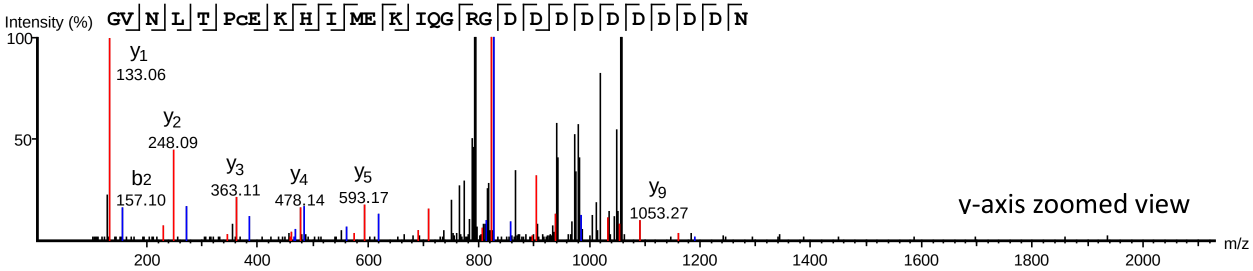
Intensity (%)
GV N L T PcE K H I ME K IQG RG D D D D D D D D D N
100
50
y1 133.06
y2 248.09
b2 157.10
y3 363.11
y4 478.14
y5 593.17
y9 1053.27
y-axis zoomed view
200 400 600 800 1000 1200 1400 1600 1800 2000
m/z

5.

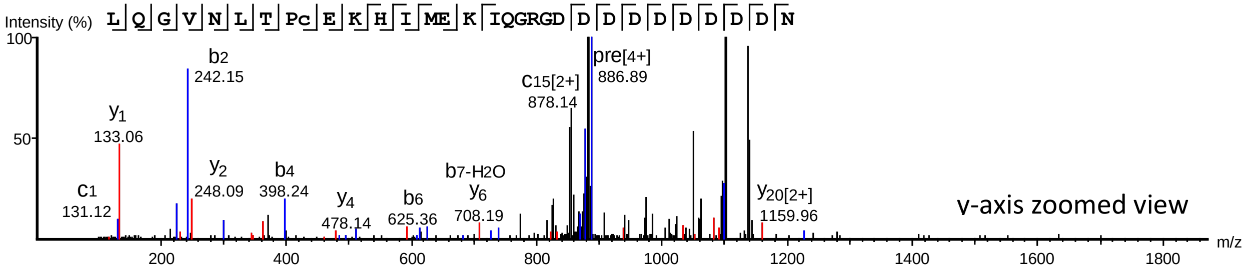
Intensity (%)
L Q G V N L T Pc E K H I ME K IQGRGD D D D D D D D D N
100
50
b2 242.15
y1 133.06
c1 131.12
y2 248.09
b4 398.24
y4 478.14
b6 625.36
b7-H2O
y6 708.19
c15[2+] 878.14
pre[4+] 886.89
y20[2+] 1159.96
y-axis zoomed view
200 400 600 800 1000 1200 1400 1600 1800
m/z

6.

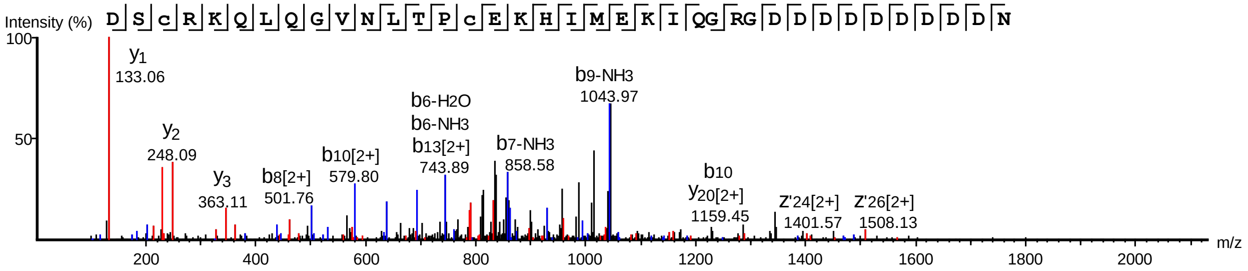
Intensity (%)
D S c R K Q L Q G V N L T P c E K H I M E K I QG RG D D D D D D D D D N
100
50
y1 133.06
y2 248.09
y3 363.11
b8[2+] 501.76
b10[2+] 579.80
b6-H2O
b6-NH3
b13[2+] 743.89
b7-NH3 858.58
b9-NH3 1043.97
b10
y20[2+] 1159.45
Z'24[2+] 1401.57
Z'26[2+] 1508.13
200 400 600 800 1000 1200 1400 1600 1800 2000
m/z

7.

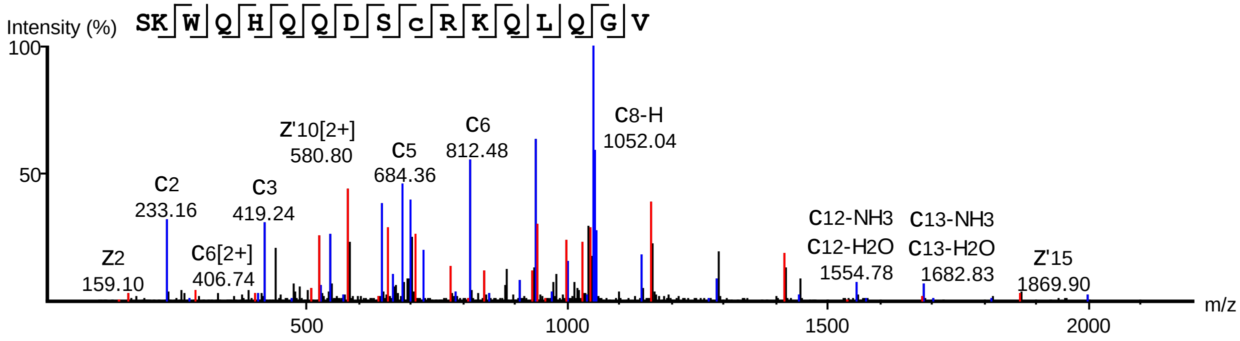
Intensity (%)
SK W Q H Q Q D S c R K Q L Q G V
100
50
z2 159.10
c2 233.16
c6[2+] 406.74
c3 419.24
z'10[2+] 580.80
c5 684.36
c6 812.48
c8-H 1052.04
c12-NH3
c12-H2O
1554.78
c13-NH3
c13-H2O
1682.83
z'15 1869.90
500 1000 1500 2000
m/z

8.

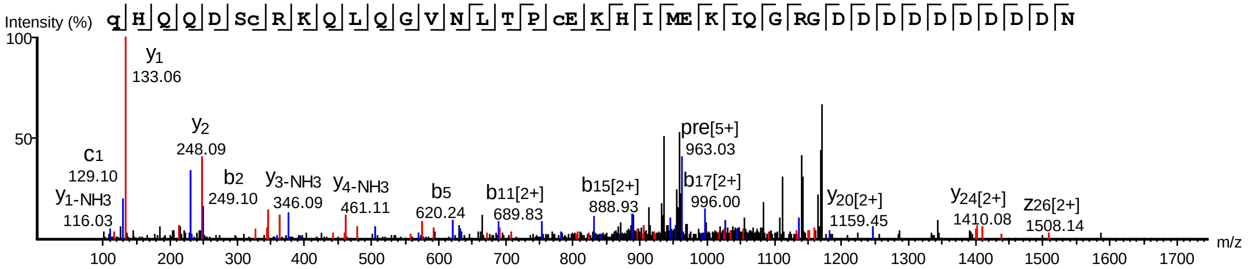
Intensity (%)
q H Q Q D Sc R K Q L Q G V N L T P cE K H I ME K IQ G RG D D D D D D D D D N
100
50
y1 133.06
y2 248.09
c1 129.10
y1-NH3 116.03
b2 249.10
y3-NH3 346.09
y4-NH3 461.11
b5 620.24
b11[2+] 689.83
b15[2+] 888.93
pre[5+] 963.03
b17[2+] 996.00
y20[2+] 1159.45
y24[2+] 1410.08
z26[2+] 1508.14
m/z
100 200 300 400 500 600 700 800 900 1000 1100 1200 1300 1400 1500 1600 1700

9.

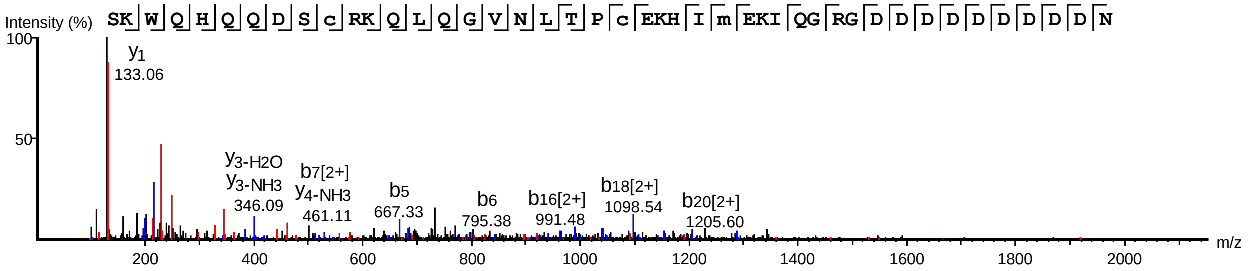
Intensity (%)
SK W Q H Q Q D S c RK Q L Q G V N L T P c EKH I m EKI QG RG D D D D D D D D D N
100
50
y1 133.06
y3-H2O
y3-NH3 346.09
b7[2+]
y4-NH3 461.11
b5 667.33
b6 795.38
b16[2+] 991.48
b18[2+] 1098.54
b20[2+] 1205.60
m/z
200 400 600 800 1000 1200 1400 1600 1800 2000

10.

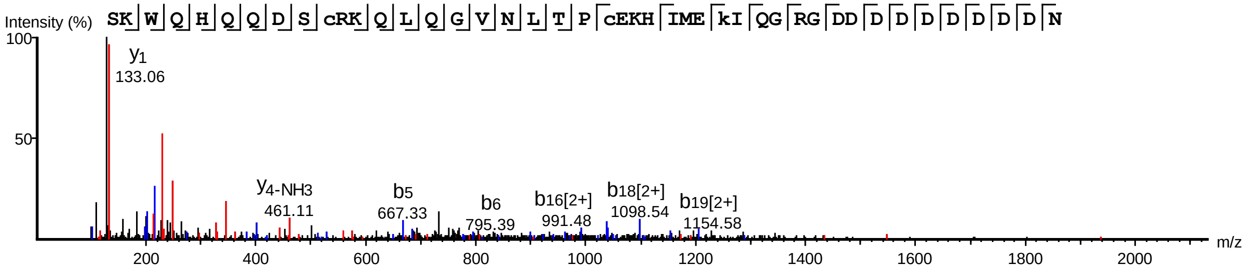
Intensity (%)
SK W Q H Q Q D S cRK Q L Q G V N L T P cEKH IME kI QG RG DD D D D D D D D N
100
50
y1 133.06
y4-NH3 461.11
b5 667.33
b6 795.39
b16[2+] 991.48
b18[2+] 1098.54
b19[2+] 1154.58
m/z
200 400 600 800 1000 1200 1400 1600 1800 2000

11.

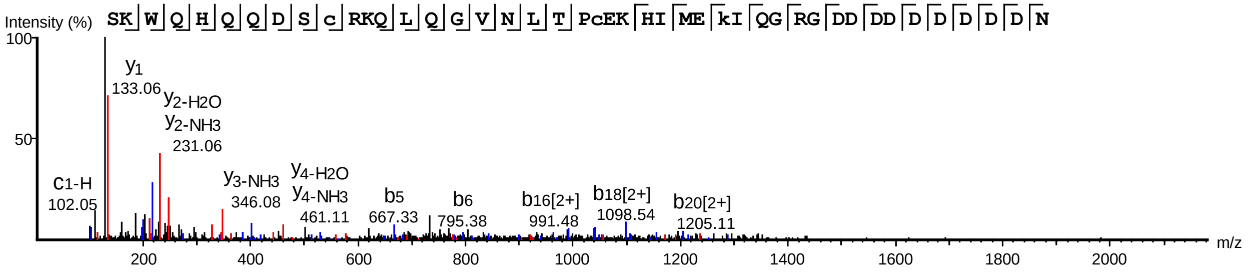
Intensity (%)
SK W Q H Q Q D S c RKQ L Q G V N L T PcEK HI ME kI QG RG DD DD D D D D D N
100
50
y1 133.06
y2-H2O
y2-NH3 231.06
c1-H 102.05
y3-NH3 346.08
y4-H2O
y4-NH3 461.11
b5 667.33
b6 795.38
b16[2+] 991.48
b18[2+] 1098.54
b20[2+] 1205.11
m/z
200 400 600 800 1000 1200 1400 1600 1800 2000

12.

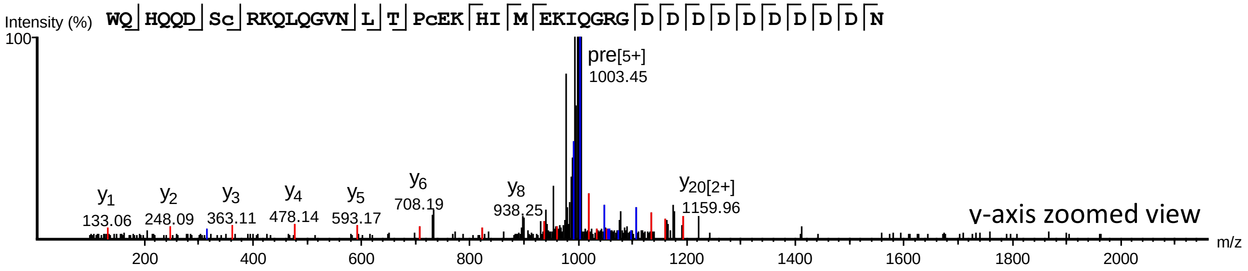

Intensity (%)
WQ HQQD Sc RKQLQGVN L T PcEK HI M EKIQGRG D D D D D D D D D N
100
pre[5+]
1003.45
y1
133.06
y2
248.09
y3
363.11
y4
478.14
y5
593.17
y6
708.19
y8
938.25
y20[2+]
1159.96
y-axis zoomed view
200 400 600 800 1000 1200 1400 1600 1800 2000
m/z

13.

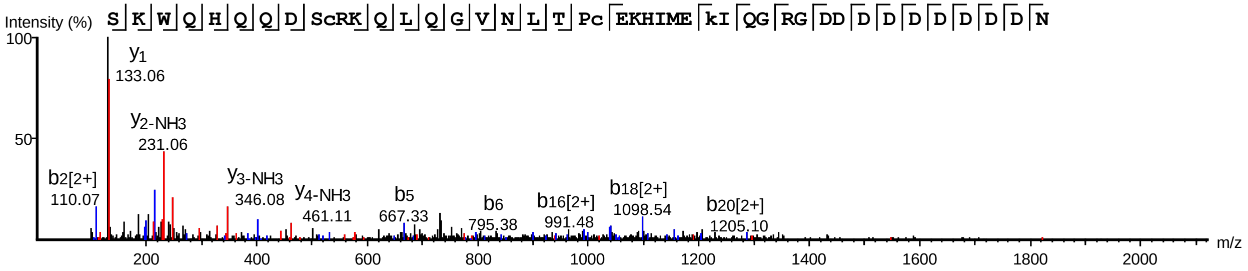

Intensity (%)
S K W Q H Q Q D ScRK Q L Q G V N L T Pc EKHIME kI QG RG DD D D D D D D D N
100
50
y1
133.06
y2-NH3
231.06
b2[2+]
110.07
y3-NH3
346.08
y4-NH3
461.11
b5
667.33
b6
795.38
b16[2+]
991.48
b18[2+]
1098.54
b20[2+]
1205.10
200 400 600 800 1000 1200 1400 1600 1800 2000
m/z

14.

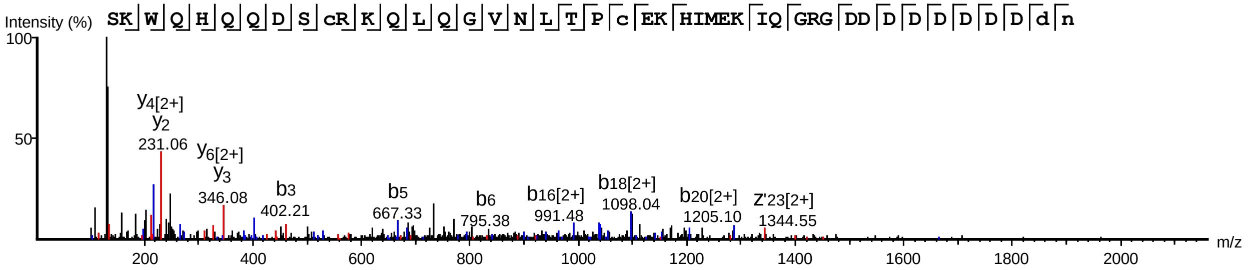

Intensity (%)
SK W Q H Q Q D S cR K Q L Q G V N L T P c EK HIMEK IQ GRG DD D D D D D D d n
100
50
y4[2+]
y2
231.06
y6[2+]
y3
346.08
b3
402.21
b5
667.33
b6
795.38
b16[2+]
991.48
b18[2+]
1098.04
b20[2+]
1205.10
z'23[2+]
1344.55
200 400 600 800 1000 1200 1400 1600 1800 2000
m/z

15.

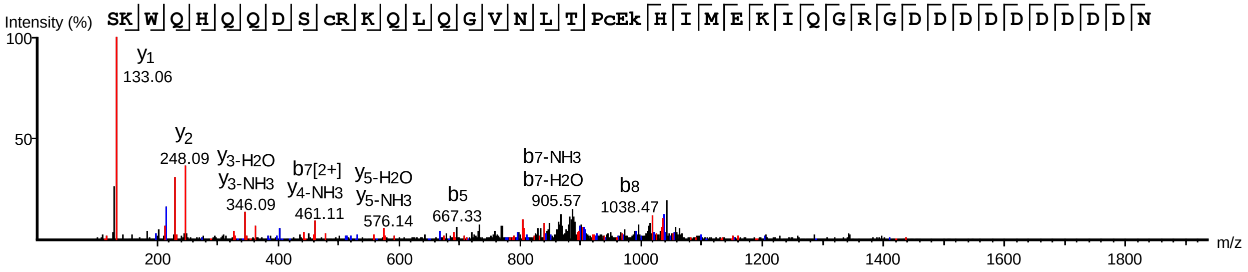

Intensity (%)
SK W Q H Q Q D S cR K Q L Q G V N L T PcEk H I M E K I Q G R G D D D D D D D D D N
100
50
y1
133.06
y2
248.09
y3-H2O
y3-NH3
346.09
b7[2+]
y4-NH3
461.11
y5-H2O
y5-NH3
576.14
b5
667.33
b7-NH3
b7-H2O
905.57
b8
1038.47
200 400 600 800 1000 1200 1400 1600 1800
m/z

16.

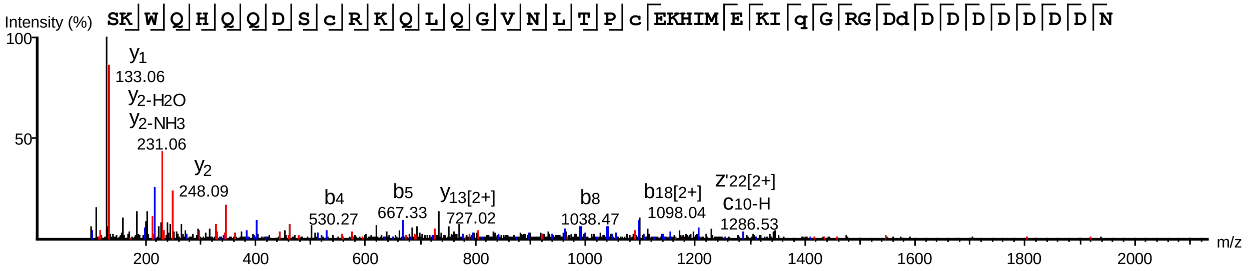
Intensity (%)
SK W Q H Q Q D S c R K Q L Q G V N L T P c EKHIM E KI q G RG Dd D D D D D D D N
y1 133.06
y2-H2O
y2-NH3 231.06
y2 248.09
b4 530.27
b5 667.33
y13[2+] 727.02
b8 1038.47
b18[2+] 1098.04
Z'22[2+]
C10-H 1286.53
m/z

17.

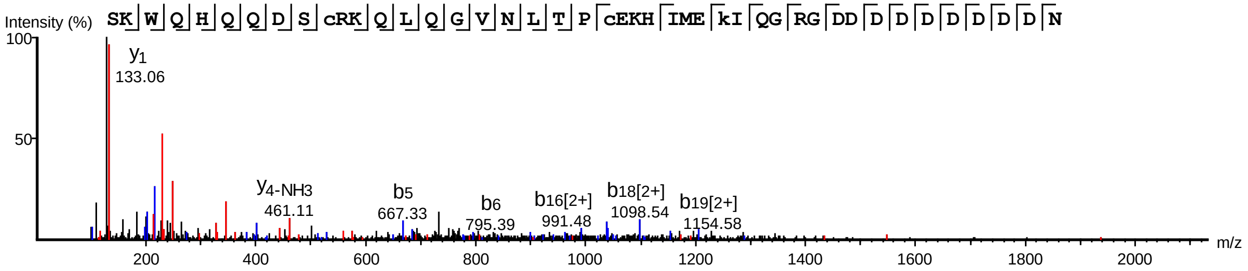
Intensity (%)
SK W Q H Q Q D S cRK Q L Q G V N L T P cEKH IME kI QG RG DD D D D D D D D N
y1 133.06
y4-NH3 461.11
b5 667.33
b6 795.39
b16[2+] 991.48
b18[2+] 1098.54
b19[2+] 1154.58
m/z

18.

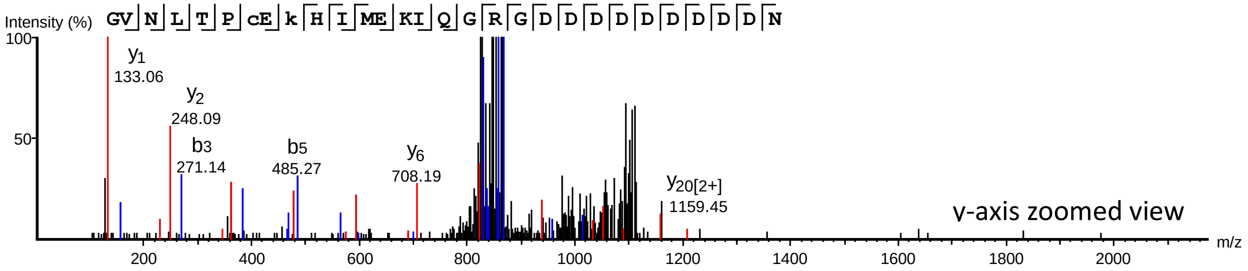
Intensity (%)
GV N L T P cE k H I ME KI Q G R G D D D D D D D D D N
y1 133.06
y2 248.09
b3 271.14
b5 485.27
y6 708.19
y20[2+] 1159.45
y-axis zoomed view
m/z

19.

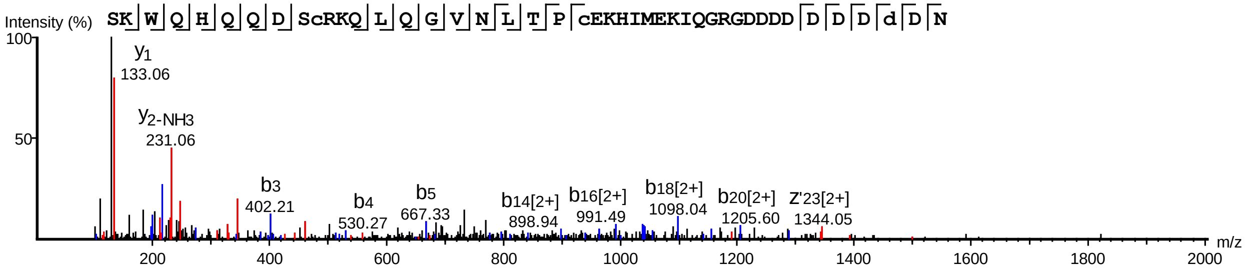
Intensity (%)
SK W Q H Q Q D ScRKQ L Q G V N L T P cEKHIMEKIQGRGDDDD D D D d D N
100
50
y1 133.06
y2-NH3 231.06
b3 402.21
b4 530.27
b5 667.33
b14[2+] 898.94
b16[2+] 991.49
b18[2+] 1098.04
b20[2+] 1205.60
z'23[2+] 1344.05
200 400 600 800 1000 1200 1400 1600 1800 2000
m/z

20.

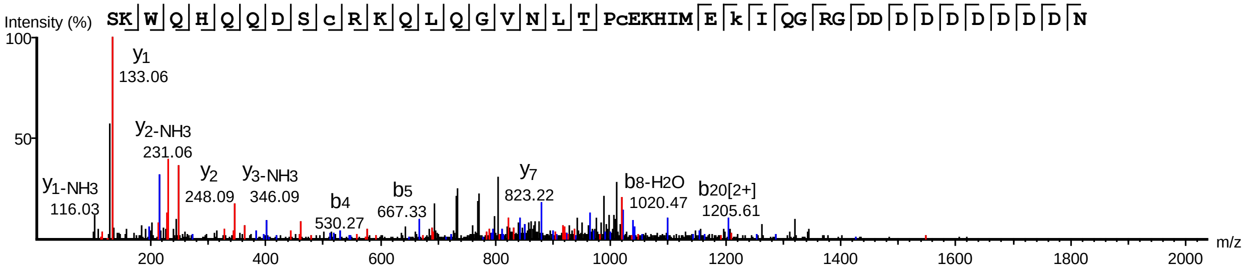
Intensity (%)
SK W Q H Q Q D S c R K Q L Q G V N L T PcEKHIM E k I QG RG DD D D D D D D D N
100
50
y1 133.06
y2-NH3 231.06
y1-NH3 116.03
y2 248.09
y3-NH3 346.09
b4 530.27
b5 667.33
y7 823.22
b8-H2O 1020.47
b20[2+] 1205.61
200 400 600 800 1000 1200 1400 1600 1800 2000
m/z

21.

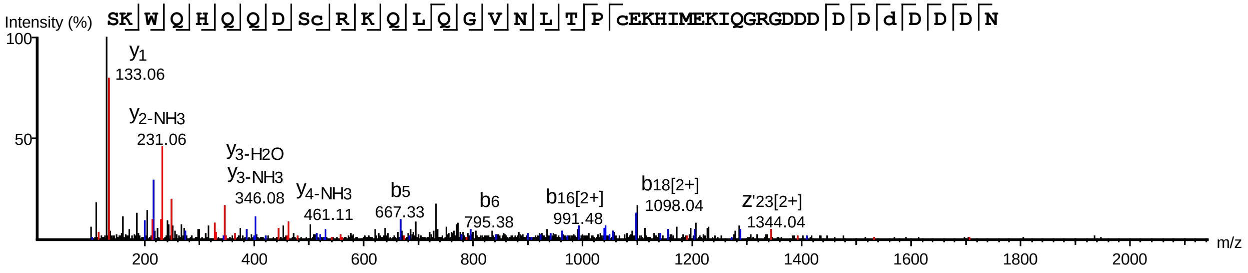
Intensity (%)
SK W Q H Q Q D Sc R K Q L Q G V N L T P cEKHIMEKIQGRGDDD D D d D D D N
100
50
y1 133.06
y2-NH3 231.06
y3-H2O
y3-NH3 346.08
y4-NH3 461.11
b5 667.33
b6 795.38
b16[2+] 991.48
b18[2+] 1098.04
z'23[2+] 1344.04
200 400 600 800 1000 1200 1400 1600 1800 2000
m/z

22.

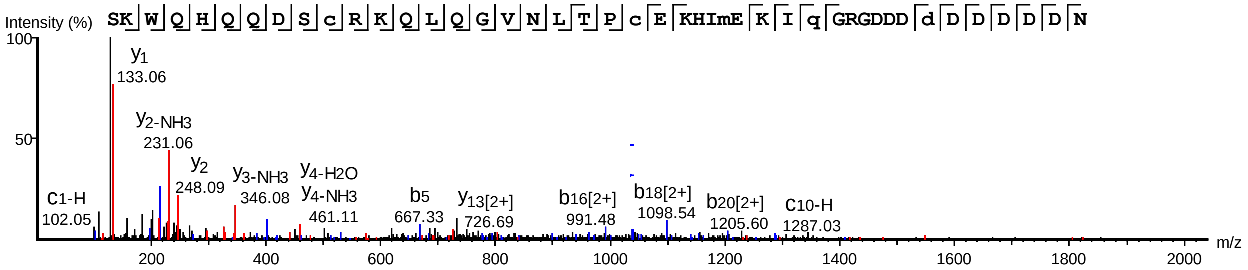

Intensity (%)
SKWQHQQDScRKQLQGVNLTPcEKHImEKIqGRGDDDdDDDDDN
100
50
y1 133.06
y2-NH3 231.06
y2 248.09
y3-NH3 346.08
y4-H2O
y4-NH3 461.11
c1-H 102.05
b5 667.33
y13[2+] 726.69
b16[2+] 991.48
b18[2+] 1098.54
b20[2+] 1205.60
c10-H 1287.03
200
400
600
800
1000
1200
1400
1600
1800
2000
m/z

23.

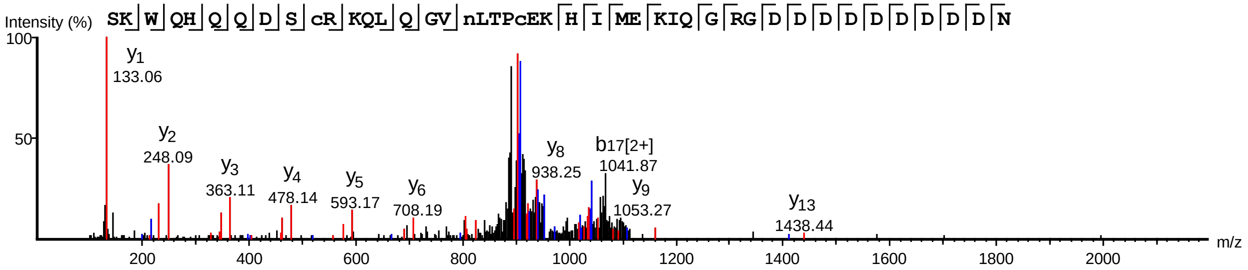

Intensity (%)
SKWQHQQDScRKQLQGVnLTPcEKHIMEKIQGRGDDDDDDDDDN
100
50
y1 133.06
y2 248.09
y3 363.11
y4 478.14
y5 593.17
y6 708.19
y8 938.25
b17[2+] 1041.87
y9 1053.27
y13 1438.44
200
400
600
800
1000
1200
1400
1600
1800
2000
m/z

24.

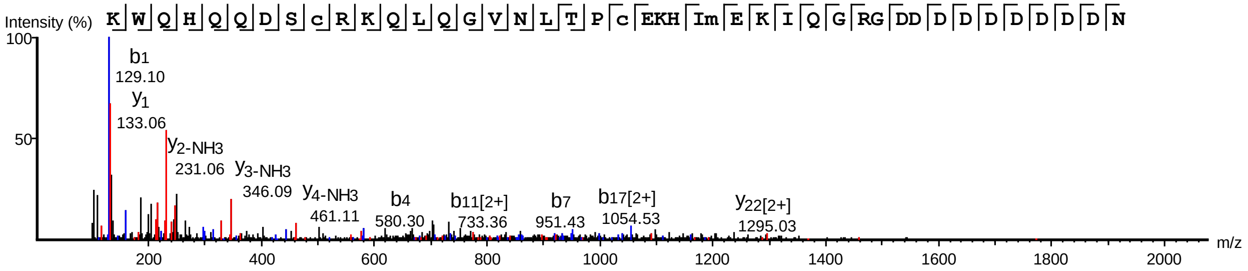

Intensity (%)
KWQHQQDScRKQLQGVNLTPcEKHImEKIQGRGDDDDDDDDDN
100
50
b1 129.10
y1 133.06
y2-NH3 231.06
y3-NH3 346.09
y4-NH3 461.11
b4 580.30
b11[2+] 733.36
b7 951.43
b17[2+] 1054.53
y22[2+] 1295.03
200
400
600
800
1000
1200
1400
1600
1800
2000
m/z

25.

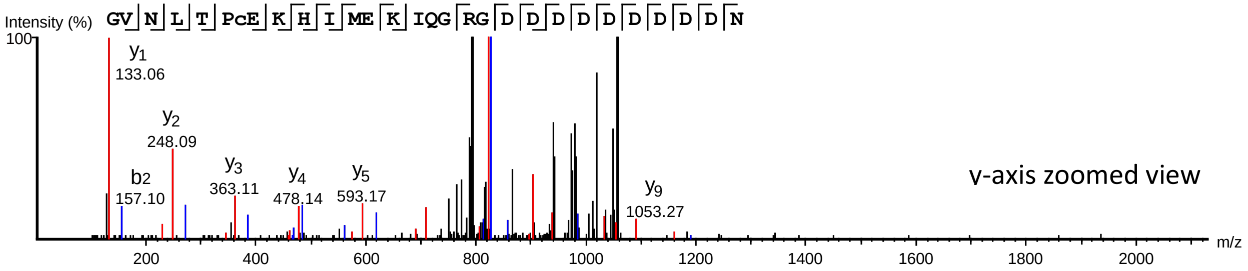

Intensity (%)
100
GV N L T PcE K H I ME K IQG RG D D D D D D D D D N
y1
133.06
y2
248.09
b2
157.10
y3
363.11
y4
478.14
y5
593.17
y9
1053.27
y-axis zoomed view
200
400
600
800
1000
1200
1400
1600
1800
2000
m/z

Supplemental information of annotated MS/MS spectra list 2: Annotated MS/MS spectra for all the identified peptides listed in Table 2. The annotated MS/MS spectra of the identified peptides in the following table are showed in subsequent pages. They are arranged by the ID# in the first column of the table. Modified residues are highlighted in the sequence shown in each annotated spectrum by lowercased letters.

Peptide	10lgP[a]	Mass	ppm	m/z	z	#Spec[b]	Side chain modifications
1. SKWQHQQDSCRKQLQGVNLTPCEKHIMEKIQGRGDDDDDDDDDD	200.00	5241.34	0.1	874.5645	6	37	EA; CEL (Lys24); CML (Lys29)
2. SKWQHQQDSCRKQLQGVNLTPCEKHIMEKIQGRGDDDDDDDDDD	90.21	5241.34	0.1	874.5645	6	16	EA; CML (Lys24); CEL (Lys29)
3. KWQHQQDSCRKQLQGVNLTPCEKHIMEKIQGRGDDDDDDDDDD	81.25	5154.31	-3.3	860.0562	6	2	EA; CEL (Lys24); CML (Lys29)
4. SKWQHQQDSCRKQLQGVNLTPCEKHIMEKIQGRGDDDDDDDDDD	45.57	5255.36	-3.1	876.8976	6	3	EA; CEL(Lys24, 29)
5. SKWQHQQDSCRKQLQGVNLTPCEKHIMEKIQGRGDDDDDDDDDDN	39.28	5283.36	3.3	881.5708	6	2	EA; CML (Lys 29)
6. SKWQHQQDSCRKQLQGVNLTPCEKHIMEKIQGRGDDDDDDDDDDN	43.46	5369.40	0.1	895.9075	6	1	EA; CEL (Lys24, Lys29)

1.

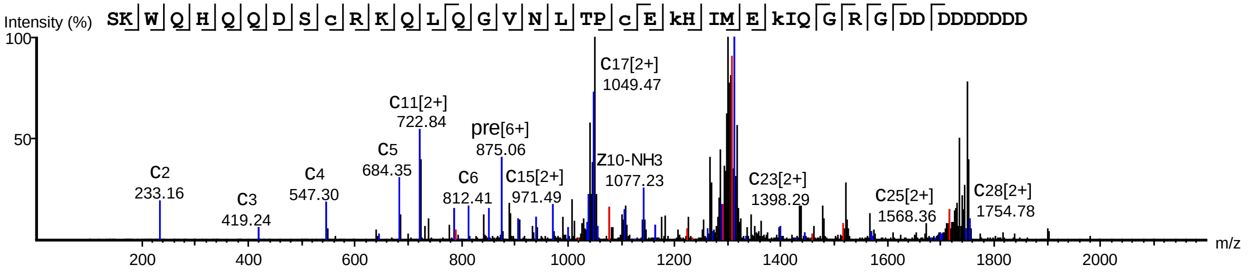

2.

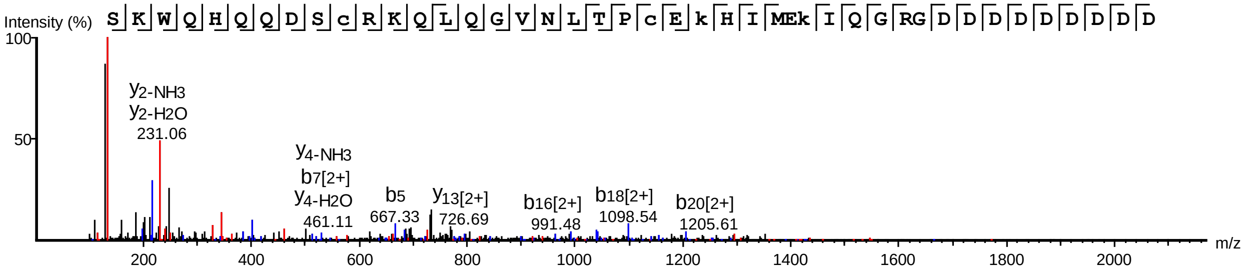

3.

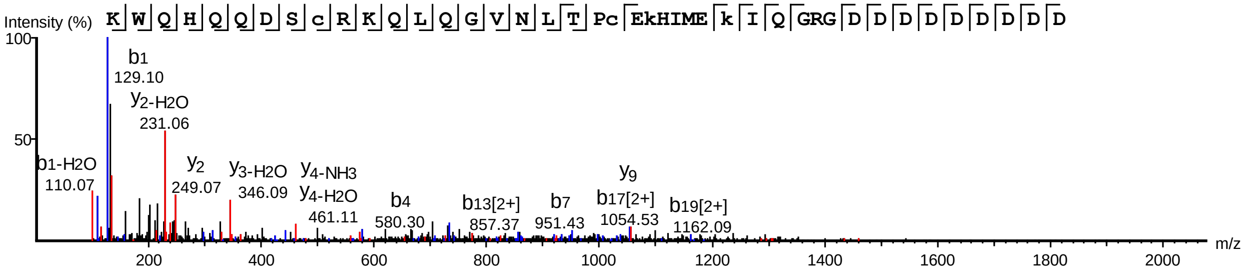
Intensity (%)
K W Q H Q Q D S c R K Q L Q G V N L T Pc EkHIME k I Q GRG D D D D D D D D
100
50
b1
129.10
y2-H2O
231.06
b1-H2O
110.07
y2
249.07
y3-H2O
346.09
y4-NH3
y4-H2O
461.11
b4
580.30
b13[2+]
857.37
b7
951.43
y9
b17[2+]
1054.53
b19[2+]
1162.09
200
400
600
800
1000
1200
1400
1600
1800
2000
m/z

4.

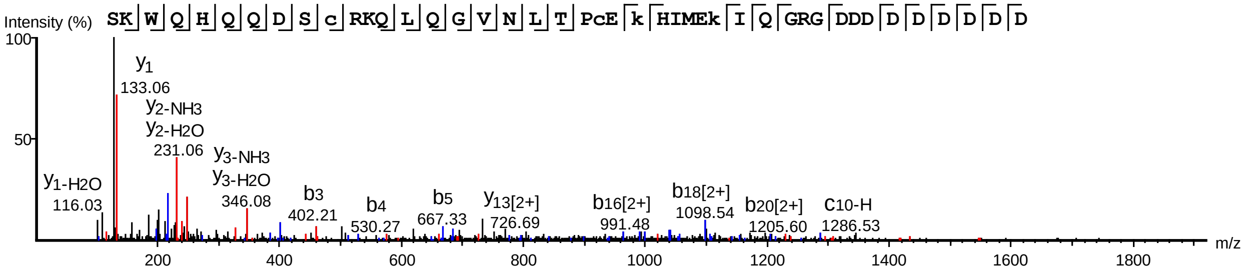
Intensity (%)
SK W Q H Q Q D S c RKQ L Q G V N L T PcE k HIMEk I Q GRG DDD D D D D D D
100
50
y1
133.06
y2-NH3
y2-H2O
231.06
y1-H2O
116.03
y3-NH3
y3-H2O
346.08
b3
402.21
b4
530.27
b5
667.33
y13[2+]
726.69
b16[2+]
991.48
b18[2+]
1098.54
b20[2+]
1205.60
c10-H
1286.53
200
400
600
800
1000
1200
1400
1600
1800
m/z

5.

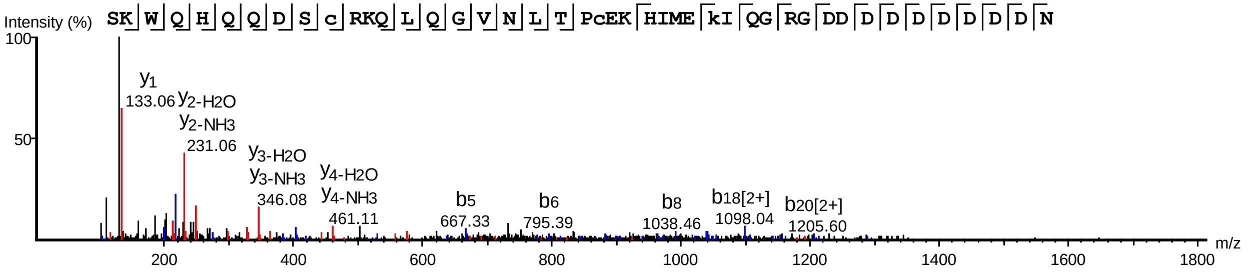
Intensity (%)
SK W Q H Q Q D S c RKQ L Q G V N L T PcEK HIME kI QG RG DD D D D D D D D N
100
50
y1
133.06
y2-H2O
y2-NH3
231.06
y3-H2O
y3-NH3
346.08
y4-H2O
y4-NH3
461.11
b5
667.33
b6
795.39
b8
1038.46
b18[2+]
1098.04
b20[2+]
1205.60
200
400
600
800
1000
1200
1400
1600
1800
m/z

6.

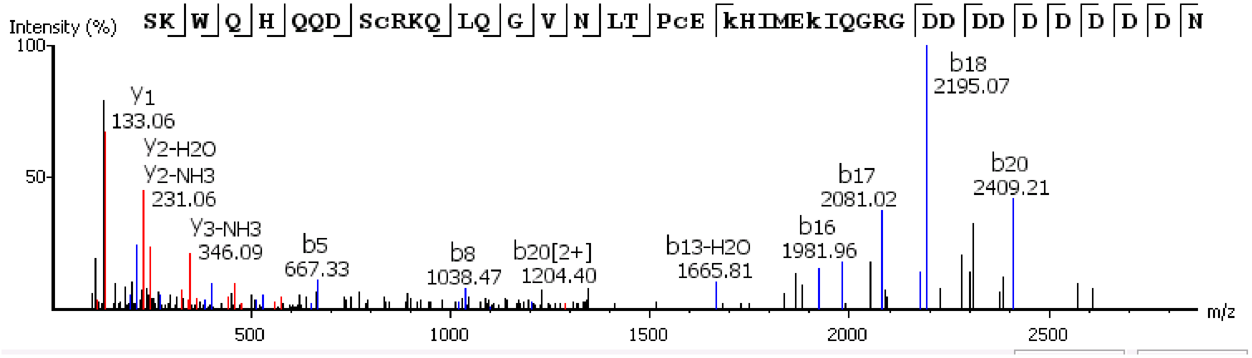
Intensity (%)
SK W Q H QQD ScRKQ LQ G V N LT PcE kHIMEkIQGRG DD DD D D D D D N
100
50
y1
133.06
y2-H2O
y2-NH3
231.06
y3-NH3
346.09
b5
667.33
b8
1038.47
b20[2+]
1204.40
b13-H2O
1665.81
b16
1981.96
b17
2081.02
b18
2195.07
b20
2409.21
500
1000
1500
2000
2500
m/z
